# Nurse training to enhance adherence counselling for HIV-tuberculosis coinfection in South Africa: Integrative review

**DOI:** 10.4102/curationis.v47i1.2557

**Published:** 2024-11-15

**Authors:** Victoire Ticha, Million Bimerew, Rene D. Phetlhu

**Affiliations:** 1 Department of Nursing, Faculty of Community and Health Sciences, University of the Western Cape, Cape Town, South Africa; 2 Department of Nursing, Faculty of Health Sciences, Sefako Makgatho Health Sciences University, Pretoria, South Africa

**Keywords:** attributes, training programme development, HIV, TB, coinfection, adherence counselling, nurse

## Abstract

**Background:**

South Africa has seen strides in reducing HIV and tuberculosis (TB); however, adherence counselling for people living with HIV (PLHIV) coinfected with TB remains a challenge, particularly in specific sub-districts like Cape Town. Understanding the attributes of existing training programmes is crucial.

**Objectives:**

This study explored attributes of training programme development for nurses and other health professionals to enhance adherence counselling for PLHIV coinfected with TB in Cape Town.

**Method:**

An integrative literature review was conducted in five steps following PRISMA guidelines. Electronic searches encompassed multiple databases: COCHRANE, PsycINFO, PUBMED, ENMBASE, Science Direct, SCOPUS, SocINDEX, Academic Search Complete, Eric, SABINET, Health Resources and World Health Organization Global Health Library Regional Indexes. Inclusion criteria encompassed English language, peer-reviewed full-text studies on training programme development, qualitative and quantitative, published between January 2012 and May 2021. Exclusion criteria included non-English articles, conference proceedings and irrelevant studies. Thematic data analysis synthesised findings.

**Results:**

Three main themes emerged: participant identification, key programme content and programme implementation process, crucial for effective training programme development.

**Conclusion:**

Identifying participants, defining programme content and outlining implementation processes are pivotal in enhancing nurses’ adherence counselling skills. This approach could stabilise patient treatment adherence, potentially reducing treatment default, loss to follow-up and mortality rates.

**Contribution:**

These findings lay the groundwork for developing effective training programmes aimed at improving adherence counselling among nurses.

## Introduction

Tuberculosis (TB) is a chronic infectious disease that has represented a major health problem over the centuries, and it has accounted for more human misery, suffering and loss of earning and failure of economic and social development than any other disease (Obeagu & Onuoha 2023).

According to the World Health Organization (WHO 2018) Global Tuberculosis Report, HIV and TB coinfection is a lethal combination, each speeding the other’s progress. People living with HIV (PLHIV) and TB account for one in three HIV-related deaths across the world (Joint United Nations Protocol on HIV and AIDS 2017).

Sub-Saharan Africa is the hardest-hit region, with approximately 70% of all PLHIV coinfected with TB worldwide (Joint United Nations Programme on HIV/AIDS [UNAIDS] 2016). The Stop TB Partnership and the Global Fund to Fight AIDS, TB and Malaria launched Find, Treat All, a joint initiative to scale up the End TB response towards universal access to TB prevention and care (WHO et al. 2020). This initiative stresses the need for a multisectoral approach to addressing the specific needs of PLHIV and TB coinfection (WHO 2018).

Reports published on the HIV and TB coinfection in South Africa highlight that about 35% of deaths among the coinfected persons were because of TB disease (Massyn et al. 2016). Ending the TB epidemic by 2030 is among the health targets of the Sustainable Development Goals (SDG) (Massyn et al. 2018). Adherence to treatment remains the main challenge (Nezenega, Perimal-Lewis & Maeder 2020). This assertion is confirmed in the South African Adherence Guidelines for HIV, TB and noncommunicable diseases (NCDs) (National Department of Health [NDoH] 2016).

The South Africa Strategy Plan (NSP) on HIV, TB and sexually transmitted infections (STIs) (The National Strategic Plan: South African Council SANAC 2018) reports that about 270 000 people became newly diagnosed with HIV, and the 2015 estimate of new TB cases were 450 000. In addition to this, the WHO (2019) reported that there were approximately 64 000 deaths caused by TB disease in South Africa in the year 2019 (WHO 2019). While the prevalence of HIV and TB is high, the Western Cape Province in 2016 recorded 74.6% cases of HIV and TB coinfection (Massyn et al. 2019), with the Cape Town District recording 81.1% of HIV and TB coinfected patients on antiretroviral therapy (ART) (Massyn et al. 2018). According to the 2019 WHO Global Tuberculosis Report: South Africa, in 2018, 104 625 HIV and TB cases were recorded. Also, less than half (49%) of the estimated 815 000 PLHIV who also have TB disease were reported to receive both HIV treatment and TB treatment (UNAIDS 2020).

Non-adherence to TB treatment and ART among HIV and TB coinfected patients is a significant barrier to successful treatment outcomes (Mazinyo et al. 2016). The burden of tablets, length of treatment, the burden of secrecy of HIV and TB condition, patient–provider relationship, patient–household interaction, alcohol intake and stigma remain inevitable in HIV and TB coinfected patients (Mandimika & Friedland 2020; Mbunyuza 2020). To combat the challenge of nonadherence, the South African Department of Health (2015) advocated for the need for adherence counselling to be conducted by an appropriately trained, mentored and supervised counsellor or healthcare worker in HIV and TB coinfected patients. While continuous adherence counselling is identified as essential to enhance medication adherence in HIV and TB coinfected patients (Southern African HIV Clinicians Society n.d.), the number of quality healthcare personnel to offer proper counselling remains a barrier (Mahtab & Coetzee 2017). Already, the clock is ticking for South Africa to reach the ambitious 2025 targets for TB and HIV laid out in the new Global AIDS Strategy for 2021–2026 (End Inequalities. End AIDS. Global AIDS Strategy 2021–2026).

Nurses have identified that TB and HIV adherence counselling remains critically inadequate with the integrated treatment of PLHIV coinfected with TB. Human immunodeficieny virus and TB adherence counselling services that nurses provide to enhance patient compliance remain insufficient. Nurses are the frontline care providers in the South African healthcare system and are pivotal in managing integrated interventions for HIV and TB coinfection (Makhado, Davhana-Maselesele & Farley 2018). According to Phetlhu et al. (2018), all categories of nurses play a role in caring for HIV and TB coinfected patients. Despite that, focussed training is directed towards registered nurses (RNs), specifically for initiation of HIV and TB treatment, and all others who encounter patients on follow-up are not adequately trained to continue with adherence counselling.

In South Africa, individuals (not qualified to undergo nurse training) are trained as community health workers (CHWs) and mentored as counsellors to conduct treatment adherence counselling (South Africa National HIV Counselling and Testing Policy Guidelines 2010). At community healthcare centres (CHC), trained counsellors offer pre- and post-test HIV counselling during screening. However, adherence counselling at the point of initiation of treatment and retention in care where nurses are the leading role players needs to be sufficiently done. Nurses are expected to continue to manage these HIV and TB coinfected patients. However, they need to be adequately trained to do adherence counselling.

This is despite the existence of the adherence guidelines on HIV, TB and NCDs with outlined strategies and procedures for implementation in South Africa. This results in poor treatment adherence, increased rate of treatment interruption and increased transmission of both infections. Therefore, a training programme is imperative to close this gap; hence, this review seeks to summarise how existing training programmes were developed. In doing so, this review highlights the different attributes when developing a training programme. Training programmes, particularly for nurses, have been reported to be effective in scaling up care and providing the individual, the patients and organisations with worthwhile benefits (Elvish et al. 2018; Simelane et al. 2018). Hence, this review aims to explore and describe attributes of a training programme development for nurses to improve adherence counselling of PLHIV coinfected with TB in South Africa. The review question was: *What are the existing adherence training programmes for nurses caring for PLHIV coinfected with TB?*

## Research methods and design

**FIGURE 1:**
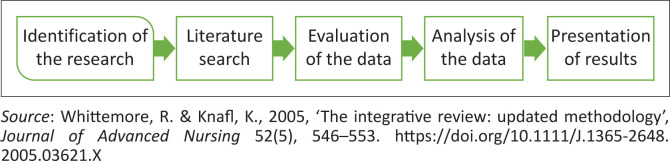
Integrative review framework.

This study used an integrative literature review design to explore and describe attributes of a training programme development for nurses to improve adherence counselling of PLHIV coinfected with TB in a selected health subdistrict, Cape Town. This review is a method to ‘summarise the literature on a specific context or content area, whereby the research is summarised, analysed, and overall conclusions are drawn’ (Whittemore 2005). Similarly, the current integrative review was considered appropriate because it plays a more significant role in evidence-based practice and policy. It can expand nursing science on the troubled issue of adherence counselling of PLHIV coinfected with TB in the CHC settings. Also, as an approach, it considers a combination of diverse methodologies. An integrative review method was considered, as this will fully provide an in-depth understanding of the attributes of concurrent training programmes that will enable the researchers to develop a training programme to improve adherence counselling of PLHIV coinfected with TB for nurses offered at the CHC level. To achieve the aim of this review, a five-step integrative review framework by Whittemore and Knafl (2005) was employed, as presented in Figure 1.

### Identification of the research problem

Considering the aforementioned background, it is clear that there exists sufficient literature on the phenomenon in question. The adherence guidelines for HIV, TB and NCDs (2016) report that the NDoH in South Africa compiled a comprehensive adherence guidelines’ document for HIV, TB and NCDs with strategies and procedures to improve linkage, adherence and retention in care. These guidelines place adherence counselling as a core to remove the barriers to adherence. However, its implementation needs to be more adequate. The guidelines clearly highlight that these are mere guidelines, and each province needs to develop ways to facilitate adherence, in this case, among PLHIV coinfected with TB. According to adherence guidelines for HIV, TB and NCDs (2016), critical structures provide related barriers, including poor patient-provider communication, lack of adequate health education, level of engagement and empathy towards patients as well as inadequate training of staff on counselling, particularly adherence counselling. These barriers are reported from a national perspective, which includes the Western Cape as one of the provinces in South Africa.

Despite the reports on structural barriers, there are no training programmes that sufficiently train all cadre of nurses on adherence counselling, albeit the need for them to implement or use proposed strategies in the adherence guidelines for HIV, TB and NCDs. Hence, there is a need to carry out an integrative literature review to assess the existing attributes in previous training programmes. The variables in this review and the population comprised of articles retrieved from the initial search using the search strategy described as follows.

### Literature search

The literature search was done using keywords, and synonyms were used across all included databases with the assistance of an experienced librarian and as per database search criteria (for instance, Mesh in PubMed, descriptor in PsycArticle). The Boolean operators ‘AND’ and ‘OR’ were used to combine all concepts. The search terms for both levels were PLHIV and TB AND training programme; education AND HIV/TB, training programme AND HIV, training, AND TB; training programme AND HIV/TB, training AND medication intake, training for adherence counselling, training for treatment adherence, training programme AND implementation, training programme AND evaluation, educational OR training programme, guidelines AND medication intake, training programme and implementation. Using these terms combined, the following databases were searched: COCHRANE, PsycINFO, PUBMED, ENMBASE, Science Direct, SCOPUS, SocINDEX, Academic Search Complete, Eric, SABINET, Health Resources, the WHO Global Health Library Regional Indexes (AIM [AFRO], LILACS [AMRO/PAHO], IMEMR [EMRO], IMSEAR [SEARO], and WPRIM [WPRO]), Google Scholar, and Sage. The results were imported into Mendeley reference software for further processing. Finally, the reference lists of critical articles identified were hand-searched to identify further relevant articles (Madhani et al. 2014).

#### Inclusion and exclusion criteria

After stating the purpose of this review and formulating a straightforward review question, the researchers outlined the inclusion and exclusion criteria for this review. This was done to ensure that articles retrieved from the literature were following the set inclusion and exclusion criteria for this integrative review. The selected studies were published in English. Some of the articles were peer-reviewed and full-texted. Some were from international articles like WHO. Also, there was no restriction regarding the setting or the country where the studies were conducted. Grey literature in the form of reports was also included. All studies focussed on training programme development. These studies were published between 01 January 2012 and 31 May 2021. This period was purposively selected, as this duration provided an extensive period to assess primary articles on training programme development. The researchers anticipated that this period would provide relevant reports and recent evidence related to the topic if they existed. The exclusion criteria included articles in the press, conference proceedings, articles that were not relevant to the aim of the review, and non-English articles. Two reviewers performed article selection by reading the titles and abstracts of all the resulting studies and sequentially excluding records according to the inclusion criteria.

#### Descriptors for the search

Overall, 769 articles were screened across all databases and imported into Mendeley reference manager software. The synthesis of the integrative review consists of the overall quality of the selected studies for the review and the discussion of the answers to the review based on the analysis. After the initial search, all duplicates and irrelevant articles in the Mendeley database were removed, and the search data were exported to an Excel spreadsheet. The selected studies were primarily interventional studies. Then, all irrelevant articles and reports (693) were deleted.

**FIGURE 2:**
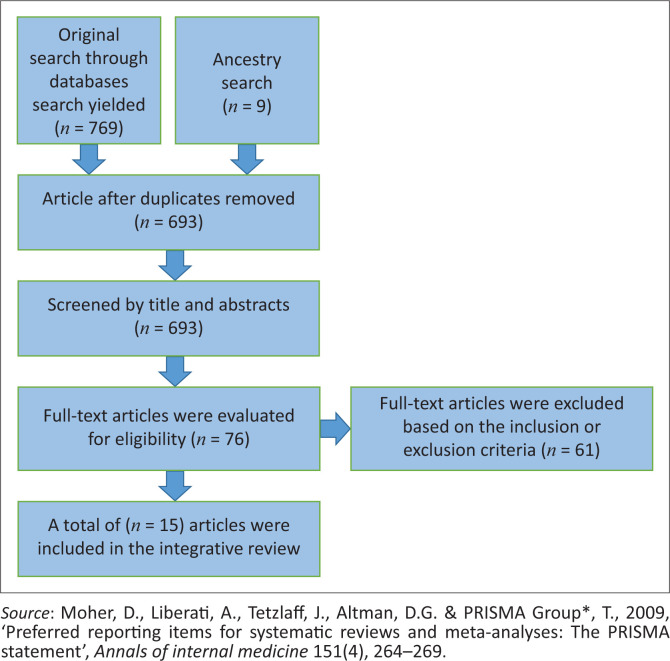
PRISMA flow diagram depicting the selection process and final number of selected articles in this study.

The remaining 76 articles were exported into a rich text table format for abstract screening. The abstracts of the 76 remaining articles were assessed, and a further 61 articles were removed. The remaining 15 articles were evaluated for quality assessment using the Joanna Briggs Institute (JBI) critical appraisal tool. About the review question, six qualitative articles from the selected studies addressed training programme or course development (Henoch et al. 2015; Hinneburg et al. 2020; Malan et al. 2016; Plowright et al. 2018; Van der Giessen et al., 2020), four quantitative articles (Driessche et al. 2009; Elvish et al. 2018; Represas-Represas et al. 2013; Van Der Giessen et al. 2020), three nonresearch documents (Simelane et al. 2018; WHO 2005, 2015) and two mixed-method articles (Couper et al. 2018; Uwimana et al. 2012). Themes were generated from the selected articles for the review. If the title and abstract met the inclusion criteria, the full text of the articles was read to determine if they met the inclusion criteria. A total of 15 articles were included as the final sample in this review. The result of the search process is depicted in Figure 2.

Articles for review were further critically appraised using the JBI appraisal tool (*[PDF] 2017 Guidance for the Conduct of JBI Scoping Reviews* n.d.).

It is a list of 10 questions with three possible answers (Yes, No and Unclear). Although the tool did not have a score, the researchers, in line with other researchers (Ma et al. 2020; Semegni et al. 2021) decided to give a score of 1 to all questions with a ‘Yes’ for an answer and 0 to all questions with a ‘No’ for an answer, and 0.5 for all questions with an ‘Unclear’ answer. The studies with scores between 0 and 2 were considered as poor quality. The scores between 3 and 4 were regarded as fair quality, and scores between 6 and 10 were good quality. In the end, 15 articles were assessed for quality and were good; hence, they were included in the review as indicated in Table 1.

**TABLE 1:** Summary of articles included in the review.

Author(s), year, type of study and country	Research aim	Setting and sample size	Data collection method	Results	Conclusion	Scoring
World Health Organization WHO (2005) Geneva	To design a training designed for tuberculosis (TB) and HIV managers operating at a national or subnational level who are responsible for planning, organising, implementing and evaluating activities within TB control programmes and/or HIV/AIDS programmes.	Setting not reported Physicians (doctors)	A training designed for TB and HIV managers	Guide for facilitators (TB and HIV managers)	A training designed for TB and HIV managers	8.5 (Good)
Driessche et al. (2009) Democratic Republic of Congo	To develop training materials to promote HIV services for TB patients	Setting not reported Sample size; 67 healthcare workers (HCWs)	Questionnaires were administered pre- and post-training, by correlating post-training results of HCWs with the centre’s HIV testing acceptance rates, and through participatory observations at the time of on-site supervisory visits and monthly meetings.	Pre-training assessment identified gaps in basic knowledge of HIV epidemiology; the link between TB and HIV; interpretation of CD4 counts; prevention and management of OIs; and occupational post-exposure prophylaxis (PEP). Opinions on patients’ rights and confidentiality varied. Mean test results increased from 72% pre-training to 87% post-training (*p* < 0.001), testing acceptance rates (*p* = 0.01). On-site supervisory visits and monthly meetings promoted staff motivation, participatory problem solving and continuing education. Training was also used as an opportunity to improve patient-centred care and HCWs’ communication skills.	Important issues regarding HIV epidemiology and PEP remained poorly understood post-training. Mean post-training scores of the clinic’s HCWs were significantly correlated with the centre’s HIV	7.5 (Good)
Uwimana et al. (2012) KwaZulu-Natal: South Africa Intervention study	To describe a participatory approach to implement and evaluate ways to integrate and train community care workers (CCWs) to enhance collaborative TB/HIV/PMTCT activities, and home-based HIV counselling and testing (HCT) at the community level.	In Sisonke District, a rural district of KwaZulu-Natal (KZN) province in South Africa 89 community care workers (CCWs)	Survey	The baseline HH survey revealed that, of 3012 household (HH) members visited by CCWs in 2008, 21% were screened for TB symptoms, 7% were visited for TB adherence support, 2% for ART adherence, and 1.5% were counselled on infant feeding options. A total of 89 CCWs were trained. Data show that during the study period in IC, 684 adults were offered HCT by CCWs, 92% accepted HCT and tested and 7% tested HIV-positive and were referred to the clinic for further care. Of 3556 adults served in intervention cluster (IC), 44% were screened for TB symptoms and 32% for symptoms of sexually transmitted infections (STIs) and 37% of children were traced as TB contact. Out of 6226 adults served followed by the control cluster (CC), 10% were screened for TB symptoms and 7% for STI symptoms. The differences in uptake of services between IC and CC were statistically significant (*p* < 0.05).	The findings of this study suggest a higher uptake of TB and STI symptoms screening, TB contact tracing and home-based HCT in the intervention clusters. This study suggests that upskilling CCWs could be one avenue to enhance TB/HIV case finding, TB contact tracing and linkages to care.	7.5 (Good)
Couper et al. (2018) Descriptive study, cross-sectional Kenya, Nigeria, South Africa and Uganda	To review the training and curricula of mid-level health workers to ascertain areas of improvement	Rural and urban government district health facilities in Kenya, Nigeria, South Africa and Uganda *N = 421* district managers and *N = 975* mid-level health workers (MLWs)	Self-administered questionnaires and in-depth interviews	Quantitative results: commonalities in the scope of practice and training programmes across all four countries. Significant concerns about skills gaps and quality of training were raised. Qualitative results: most health workers and district managers indicated that training methods needed updating with additional skills offered. Participants wanted their training to include more practical procedures that could be lifesaving.	-	6.0 (Good)
World Health Organization (2015) Geneva	To assist facilitators in training CHWs and CVs in integrating community-based TB services into their work.	All CHWs and community volunteers (CVs)	A training guide for all CHWs and CVs	A comprehensive training step-by-step guide for CHWs and CVs by a facilitator.	-	8.5 (Good)
Plowright et al. (2018) KwaZulu-Natal, South Africa Intervention design	To develop and pilot an inexpensive (2-day) training intervention covering national government priorities: HIV/AIDS; sexually transmitted disease and TB; and women’s sexual and reproductive health and rights.	Two districts (A and B) in KwaZulu-Natal Province in South Africa 64 CHWs	Unstructured interviews	Following the training intervention, improvements in knowledge scores were seen across topics and districts. However, the CHWs assigned to the test-train schedule in one district showed high gains in knowledge prior to receiving the training. All CHWs reported high levels of satisfaction with the training and marked improvements in their confidence in advising clients. The training costs around US$48 per CHW per day and has the potential to be cost-effective if the large gains in knowledge are translated into improved field-based performance and thus health outcomes.	Training CHWs can result in large improvements in knowledge with a short intervention. However, improvements seen in other studies could be because of test ‘reactivity’. Further work is needed to measure the generalisability of our results, retention of knowledge and the extent to which improved knowledge is translated into improved practice.	7.0 (Good)
Hinneburg et al. (2020) Germany	To develop and pilot a blended learning training programme for physicians and medical students to enhance their competencies in evidence-based decision-making	Martin Luther University Halle-Wittenberg in Germany 29 healthcare professionals	Focus group interview	The participants rated the comprehensibility of the learning modules as high. However, the practical exercises (e.g. role-plays in shared decision-making) revealed that relevant subjects were insufficiently understood (e.g. the difference between the benefits and harms of a diagnostic test and its test accuracy). The interactive instructional design was appreciated.	The programme was revised iteratively according to the results. Critical health competencies increased significantly after the training.	8.0 (Good)
Simelane et al. (2018) South Africa: Cape Town	To describe the features of the Practical Approach to Care Kit (PACK) training strategy: PACK training is scaled up using a cascade model of training using educational outreach to deliver PACK to clinical teams in their health facilities in short, regular sessions.	Primary healthcare centres Primary care facilities throughout South Africa	Not specified; however, a PACK training programme for scaling up and sustaining support for health workers to improve primary care has been developed and implemented.	The PACK training programme has been carefully refined to embed the programme into everyday clinical practice.	It is considered a unique and innovative programme, significantly different to other current offerings in South Africa and other low- and middle-income countries.	7.0 (Good)
Represas-Represas et al. (2013) Spain	To analyse the short- and long-term effectiveness of a supervised training programme for performing and interpreting spirometries	Healthcare centres‡ 74 nursing staff from 26 health centres	A 10-question survey	A training programme based on theoretical and practical workshops and a supervised follow-up of pyrometry significantly improved the ability of primary healthcare professionals to carry out and interpret spirometries testing, although the quality of the test diminished over time.	-	7.0 (Good)
Henoch et al. (2015) Cape Town	Development of an existential support training programme for healthcare professionals	Nurses and other healthcare professionals	Data collection followed the steps of the Medical Research Council (MRC) framework for the development and evaluation of complex interventions which are literature review of best practices and theoretical foundations for the training program, expert consultation in existential psychology, nursing, and communication to gather insights and validate the content of the training program. Focus groups/interviews with healthcare professionals to understand their needs, challenges, and experiences related to communicating about existential issues with patients.	Nurses’ confidence in communication increased after training. The understanding of the change process was considered to be that the nurses could describe their way of communicating in terms of prerequisites, process and content. Some efforts have been made to implement the training intervention, but these require further elaboration.	Existential and spiritual issues are very important to severely ill patients, and healthcare professionals need to be attentive to such questions. Professionals must be properly prepared when patients need this communication. An evidence-based training intervention could provide such preparation. Healthcare staff were able to identify situations where existential issues were apparent, and they reported that their confidence in communication about existential issues increased after attending a short-term training programme that included reflection. In order to design a programme that should be permanently implemented, more knowledge is needed of patients’ perceptions of the quality of the healthcare staff’s existential support.	7.0 (Good)
Van der Giessen et al. (2020) The Netherlands	To develop and evaluate a training programme for healthcare professionals (breast surgeons and specialised nurses), to increase effective communication	65 breast surgeons and specialised nurses 17 hospitals in the Netherlands	Group interviews	They did not feel confident in recognising limited health literacy, and their self-efficacy to communicate effectively with these patients was low. The training programme was rated as acceptable and useful. Healthcare professionals lack the confidence to effectively communicate with these patients with limited health literacy or migrant backgrounds.	The training programme offers opportunities to improve communication about referral to breast cancer genetics counselling.	7.5 (Good)
Malan et al. (2016) South Africa	To evaluate a training programme for primary care providers to offer brief behaviour change counselling on risk factors for noncommunicable diseases in South Africa	Setting not indicated 41 primary healthcare providers	Motivational interviewing	To evaluate the effect on clinical practice of training primary care providers (PCPs) in an approach to brief behaviour change counselling (BBCC), integrating the 5As (ask, alert, assess, assist and arrange) with a guiding style derived from motivational interviewing in the South African context.	-	6.5 (Good)
Van der Giessen et al. (2020)	To examine the effect of a blended training programme (Erfo4all) for healthcare professionals on their awareness, knowledge, and self-efficacy towards communication about genetic counselling with patients with limited health literacy or a migrant background.	Dutch hospitals 59 healthcare breast surgeons 16 specialised nurses	Online questionnaire	We found a significant increase in self-assessed awareness of the prevalence and impact of limited health literacy and healthcare professionals’ self-efficacy to recognise limited health literacy and to communicate effectively with patients with limited health literacy or a migrant background. We did not find an increase in knowledge scores.	A blended training programme for healthcare professionals has the potential to improve their ability to communicate effectively about breast cancer genetic counselling with patients with limited health literacy or migrant background and offers a promising way to increase the referral rate for these groups of patients.	7.0 (Good)
Elvish et al. (2018)†§ The United Kingdom	To evaluate a second phase, roll out of a dementia care training programme for general hospital staff and to further develop two outcome scales: the confidence in dementia scale for measuring confidence in working with people with dementia and the knowledge in dementia scale for measuring knowledge in dementia	Three National Health Service (NHS) Trusts situated in North-West England *N* = 517	Self-reporting questionnaire was used	A statistically significant change was identified between pre-post training on all outcome measures. Medium- to large effect sizes were demonstrated on all outcome measures. The psychometric properties of the confidence in dementia and knowledge in dementia scales are reported.	Staff knowledge of dementia and confidence in working with people with dementia significantly increased following attendance at the training sessions	8.5 (Good)
Malan et al. (2015)	To redesign the current training for primary caregivers in South Africa, around a new model for brief behavioural change counselling that would offer a standardised approach to addressing patients’ risky lifestyle behaviours	Stellenbosch University 123 of nurses and primary care doctors	ADDIE model for design of training programme (analyse, design, develop, implementation and evaluation) Motivational interviewing	A new training programme for primary caregivers was based on a conceptual model with a guiding style derived from motivational interviewing. The programme was developed as an 8 h training programme that combined theory, modelling and simulated practice with feedback, for either clinical nurses’ practitioners or primary care doctors	-	7.0 (Good)

† , A pre-post design assessed changes following completion of the ‘Getting to Know Me’ training programme.

‡ , within the healthcare area of Vigo (Galicia, Spain).

§ , Northern West England.

Also, there was no restriction regarding the setting or the country where the studies were conducted. Grey literature in the form of reports was also included. Both qualitative and quantitative articles on training programme development were included. The exclusion criteria included articles in the press, conference proceedings, articles that were not relevant to the aim of the review, and non-English articles. Two reviewers performed article selection by reading the titles and abstracts of all the resulting studies and sequentially excluding records according to the inclusion criteria (Jackson et al. 2015).

### Data evaluation

To assess the methodological quality, the articles selected were assessed by two independent reviewers for validity prior to their inclusion in the review. Descriptive data extraction and presentation were done to extract attributes of training programme development that would assist the researcher in developing a training programme for nurses to enhance adherence counselling in the Khayelitsha health subdistrict, which was the aim of the study. All 15 studies with good methodological quality were retained, and data extraction was conducted using the JBI extraction tool. All the studies in the different articles applied a quantitative, mixed-method or qualitative approach. In terms of quality assessment, the Joanna Briggs Institute Meta-Analysis of Statistics Assessment and Review Instrument (JBI-MAStARI) was used for critical appraisal (Peters et al. 2015). This tool contains a separate appraisal checklist for each type of study design. Using this instrument, two reviewers assessed the articles independently before inclusion in the final review. Any disagreements which arose among the reviewers were resolved through discussion sessions. The JBI-MAStARI tool was used to extract the data. The data extracted included details about the author(s), country, design, aim(s), sample size, settings and the result of the review. Conclusions were also extracted. These checklists critically evaluated the study’s clarity of aims, objectives, methods and appropriateness of data analysis. It also provides a comprehensive checklist of 10 evaluation criteria (Lei et al. 2014).

Evaluation tools have been developed to assist in the critical appraisal of research studies, and they provide a template of key questions to assist in the critical appraisal of quantitative research studies (Long et al. n.d.). The methodological data extracted included the author(s), country, design, aim(s), sample size, setting, result and conclusion (Table 1).

Review articles were critically appraised using the JBI appraisal tool (Guidance for the Conduct of JBI Scoping Reviews 2017). It is a list of 10 questions with three possible answers (Yes, No and Unclear). Although the tool did not have a score, the researchers, in line with other researchers (Ma et al. 2020; Semegni et al. 2021) gave a score of 1 to all questions with a ‘Yes’ for an answer and 0 to all questions with a ‘No’ for an answer, and 0.5 for all questions with an ‘Unclear’ answer. The studies with scores between 0 and 2 were considered as poor quality. The scores between 3 and 4 were considered fair quality, and scores between 6 and 10 were good quality.

### Data analysis

The purpose of the analysis stage in the integrative review was to synthesise the evidence through coding, categorising and summarising the primary sources into an unbiased integrated conclusion about the research problem (Whittemore & Knalf 2005). As a result of the heterogeneity of the studies included in this review, a meta-analysis or meta-synthesis could not be used (Higgins et al. 2003). In this review, thematic data analysis was employed for analysing and synthesising the findings of the studies included. Thematic analysis is a broadly used, flexible method for identifying, analysing and reporting patterns within data. Moreover, this method of analysis was found to be suitable for this review because it organises the main themes or concepts across diverse literature sources (A Step-by-Step Guide to Conducting an Integrative Review 2020).

Therefore, the extracted findings of the 15 articles were synthesised using a thematic analysis approach based on significant recommendations for developing a training programme. Thematic analysis is a method for identifying, analysing and reporting patterns (themes and subthemes) within data. It minimally organises and describes your data set in (rich) detail (Braun & Clarke 2006).

Data were extracted and represented textually. To facilitate the comparison of the training attributes and interpretation, data were reduced according to specific ‘issues, variables and sample characteristics’, and then data were displayed in the form of matrices, graphs or charts to allow for comparison in order to identify patterns, themes or relationships and drawing conclusion and verification (Whittermore & Knaft 2005).

### Characteristics of the studies

A total of 15 articles met the inclusion criteria and were all included in this review. This review included qualitative (6) training reports (3) and quantitative (6) studies. All included studies alluded to training programmes. The countries associated with the articles in this review were Kenya (1), South Africa (3), Geneva (2), the Republic of Congo (1), the United Kingdom (1), Germany (1), Spain (1), the Netherlands (2), Nigeria (1), Uganda (1) and Tanzania (1).

### Ethical considerations

Ethical clearance to conduct this study was obtained from the University of the Western Cape Biomedical Research Ethics Committee (reference no. BM19/8/9) on 01 July 2021.

## Results

Interpreting the extracted data about the review question was the final phase of the integrative review. The review results are presented in a narrative format, supported with tables. The themes and subthemes that emerged from the integrative literature review analysis are shown in Table 2.

**TABLE 2:** Summary of the themes and subthemes.

Themes		Subthemes
1.	Need to identify the participant	1.1. Effective communication
2.	Key content for the programme	2.1. Required skill and knowledge in counselling 2.2. Nurse–patient relationship 2.3. Nurse confidence to effectively counsel patients considering knowledge, counselling process and counselling content 2.4. Beliefs about the patient’s behaviour that is a challenge; existential and spiritual issues 2.5. Use a conceptual model that combines the 5As: ask, alert, assess, assist and arrange
3.	Process of implementing the programme	3.1. Organise interactive tuberculosis and HIV workshops with nurses

Three main themes emerged from the analysis of this review as depicted in Table 2: (1) need to identify the participant, (2) key content for the programme and (3) process of implementing the programme. These themes and subthemes emerged as successful programme development’s most influential intervention characteristics.

### Theme 1: Need to identify the participant

In this review, the need to identify the participants for developing a training programme was identified. This attribute reflects the reality of what happens at healthcare facilities. This includes effective communication.

#### Subtheme 1.1: Effective communication

Effective communication was recommended as one of the critical attributes to be included in developing a training programme. This attribute was identified in four articles and documents (Henoch et al. 2015; Van Der Giessen et al. 2020; WHO 2015). The relevance of communication in developing a training programme was described by WHO (2015) as primary in the English language. Adequate time should be allocated for proper communication to be achieved. The WHO (2015) also highlighted that, for proper communication to be achieved, the nurse’s and patient’s tone must be free, open and respectful. Van Der Giessen et al. (2020) indicated that healthcare professionals lack the confidence to effectively communicate with patients with limited literacy; hence, the authors needed to develop and evaluate a training programme for healthcare professionals to increase effective communication. Henoch et al. (2015) reported that the effectiveness of the intervention was the boost in communication of the nurse with patients. Van Der Giessen et al. (2020) further recommended self-efficacy towards communication on counselling, using plain language and teach-back methods, hence confidence in effectively communicating with the patients.

### Theme 2: Key content for the programme

This review identified several critical contents for the programme development to improve adherence counselling. They include required skill and knowledge in counselling, and nurses’ confidence to effectively counsel patients considering knowledge, counselling process and counselling content, among others.

#### Subtheme 2.1: Required skill and knowledge in counselling

Out of the 15 articles and documents appraised, 5 highlighted that nurses should possess the required skills and knowledge in counselling. Driessche et al. (2009) and Elvish et al. (2018) recommended that nurses incorporate their self-clinical knowledge and experience in counselling and see counselling as an identified required skill to achieve adherence to counselling of patients. They further highlight that the training programme should adapt existing training materials on counselling HIV and TB patients, prevention of mother-to-child transmission and management of opportunistic infections. Simelane et al. (2018) further concur that the required skill and knowledge in counselling in a training programme should consider the nurses’ existing clinical knowledge and experience. Hinneburg et al. (2020) recommend that the required skill and knowledge in counselling should make use of comprehension of the conditions at hand that require counselling, and in this review, it would be PLHIV coinfected with TB. Elvish et al. (2018) and Plowright et al. (2018) also report that knowledge about the conditions that require adherence counselling should be included in the training programme for nurses and CHWs.

#### Subtheme 2.2: Nurse–patient relationship

The nurse–patient relationship was identified in two documents in this review and recommended as a practice for developing a training programme for PLHIV coinfected with TB. This therapeutic relationship should be practised through problem-solving exercises, open discussions, and the exchange of information between the patient and the nurse (WHO 2005). This was further elaborated by WHO (2015), which recommended that English should be the primary language of communication with the patient. In case translation is needed, it should be employed so that the patient can fully comprehend the counselling service offered by the nurse. In addition, adequate counselling time should be considered, and the nurse should display a free tone consisting of an open and respectful attitude during the counselling session. A conducive environment should be created for proper counselling service; hence, an excellent nurse–patient relationship will also be established.

#### Subtheme 2.3: Nurse confidence to effectively counsel patients considering knowledge, counselling process and counselling content

Four of the 15 articles and documents reviewed reported the importance of the nurse being confident to carry out effective counselling of HIV or TB people (Elvish et al. 2018; Henoch et al. 2015; Van Der Giessen et al. 2020). The review found that the nurse’s confidence during counselling goes a long way to enhancing counselling, which takes into consideration the nurse’s clinical knowledge and experience (Elvish et al. 2018; Simelane et al. 2018). Van Der Giessen et al. (2020) recommended that the healthcare professional (nurses included) be confident to effectively counsel these patients by understanding the counselling process and content. Therefore, this contributes to the patient’s knowledge because ‘healthcare professionals lack the confidence to effectively communicate with patients with limited health literacy or migrant background’ (Van Der Giessen et al. 2020).

Furthermore, the review suggests that the nurse’s confidence to effectively treat patients should be underpinned by the nurse’s confidence in communication with the patient (Henoch et al. 2015). Van Der Giessen et al. (2020) emphasise the use of plain language by the nurse to provide effective counselling during the counselling sessions with patients.

#### Subtheme 2.4: Beliefs about the patient’s behaviour that is a challenge; existential and spiritual issues

Beliefs about the patient’s behaviour, which is a challenge, and spirituality were some other attributes recommended in two articles (Elvish et al. 2018; Henoch et al. 2015). Beliefs around patients’ negative behaviour towards adherence counselling remain a challenge. It was therefore suggested in the review that healthcare professionals should remain abreast of this challenge which would gradually improve during counselling sessions (Elvish et al. 2018). Living with a life-threatening condition like HIV and TB means living with the knowledge that the future may be limited so that existential issues might become inevitable for the ill person and family. According to Henoch et al. (2015), spirituality is expressed through beliefs, values, traditions and practices (Puchalski et al. 2014). Therefore, the recommendation in this review was that the patient’s spiritual needs should be considered when developing a training programme (Henoch et al. 2015).

#### Subtheme 2.5: Use a conceptual model that combines the 5As: Ask, alert, assess, assist and arrange

A conceptual model of brief behaviour change counselling that combines the 5As – ask, alert, assess, assist and arrange – through a guiding style derived from the motivational interview was recommended by Malan et al. (2015) and Malan et al. (2016). These counselling training intervention attributes were used to guide the style into which each of the 5As will enhance patient-centredness; in other words, it served as a guiding spirit of allowing the healthcare professional to conduct effective counselling (Malan et al. 2016). The conceptual model that combined the 5As included the following:

- Ask: Ask what the patient already knows or wants to know.
- Alert: Provide information neutrally and provide information tailored to the patient’s needs.
- Assess: Assess the patient’s confidence to change and use scaling questions correctly.
- Assist: Provide relevant practical assistance such as educational leaflets and telephone calls.
- Arrange: Arrange for follow-up appointments and demonstrate a willingness to offer ongoing support.

### Theme 3: Process of implementing the programme

This review identified organising interactive workshops as one of the attributes of training programme development. These attributes reflect the practical and supervisory follow-up sessions in developing a training programme.

#### Subtheme 3.1: Organise interactive tuberculosis and HIV workshops with nurses

Out of the 15 articles and documents, 3 indicated coordinating an interactive workshop with the nurses as one of the recommendations for the development of a training programme (Hinneburg et al. 2020; Represas-Represas et al. 2013; WHO 2005). This review highlighted that interactive TB and HIV workshops should be included in presentations and discussions on counselling. In addition, Hinneburg et al. (2020) concurred with the workshop attribute for developing a training programme using interactive instructional design workshops. Including a practical workshop and supervised follow-up sessions in developing a training programme would significantly improve the ability of primary care professionals to carry out quality patient care with the interpretation of the spirometric testing (Represas-Represas et al. 2013).

## Discussion

This integrative review aimed to describe the attributes of existing training programmes for nurses, including those that focus on adherence counselling of PLHIV coinfected with TB and other health sectors. The three primary themes identified were linked to healthcare professionals, including nurses. The significant findings of the integrative literature review show that the development of a training programme is essential to enhance adherence counselling of PLHIV coinfected with TB and other severe health conditions like cancer, dementia, spirometry (lung function test) and mental healthcare.

The nurses are seen to be the frontline workers at the PHC level, which is the entry point for diagnosis, treatment and management of TB and multi drug resistant (MDR) TB in South Africa, and the implementation of TB policies, including infection control and counselling (Zinatsa et al. 2018), is mainly dependent on nurses. Hence, all cadre of nurses registered with the South African Nursing Council (SANC) must be able to offer comprehensive care to PLHIV coinfected with TB. This review revealed that it is essential to have training programmes in place in the health sector to enhance patient care.

### Need to identify the participant

The identified attribute of effective communication for nurses to enhance adherence counselling of PLHIV coinfected with TB in South Africa has the potential to enhance a positive treatment outcome at any point of the patient’s treatment plan, which includes, from diagnosis, treatment initiation, and a steady treatment continuation of patients on ART and TB medications reduces morbidity and mortality among persons coinfected with TB and HIV (Webb Mazinyo et al. 2016). This attribute concurs with Pascoe et al. (2020) who reported that effective communication should go hand-in-hand with the use of English and possible translation into home languages, which, in this case, will include IsiXhosa and Afrikaans given the relative abundance of health resources in English. The review findings are in accordance with the findings of Path (2009), which recommended using plain language, a teach-back method, and a free, open and respectful voice tone during adherence counselling. The findings of a study by Jarrett et al. (2019) indicate that patient-centred communication is one option for nurses to shift the power dynamic and enable PLHIV coinfected with TB in South Africa to be more actively engaged in their care. For example, nurses could communicate in a way that shares power and responsibility with patients by eliciting patient ideas or expectations. Furthermore, the review suggested that the nurse’s confidence to effectively treat patients should be underpinned by the nurse’s confidence in communication with the patient (Henoch et al. 2015). Van Der Giessen et al. (2020) emphasise the use of plain language by the nurse to gain effective counselling during the counselling sessions with the patients.

### Key content for the programme

This review established that nurses should possess the required skill and knowledge in counselling, which plays a vital role in providing well-informed adherence counselling sessions between the nurse and the patient. This finding concurs with Ngcobo et al. (2022) who reported in a review that nurses and lay counsellors were skilled in counselling these patients and practised confidentiality as they share social spaces with these patients and constantly interact with their families and neighbours. This integrative literature review also recommended that nurses incorporate their self-clinical knowledge and experience in counselling, and they should see counselling as an identified required skill to enhance adherence to counselling of PLHIV coinfected with TB, as per this study. According to Elbireer et al. (2011), nurses identified the following reasons why patients default from anti-TB treatment: old age, being male, low education level, migration for work, the perception that TB is incurable, poor knowledge about TB, low income, and poor attitude of healthcare workers.

In addition, nurse–patient relationship was recommended in this review to be included in developing a training programme. It is essential for the nurses and PLHIV coinfected with TB to create and maintain a good nurse–patient relationship. This usually occurs when the healthcare provider, the nurse in this case, or the patient feels frustrated. The feeling of frustration by some healthcare providers, the nurses in this case, could be triggered by several factors, including poor working conditions and being overworked (Vera et al. 2018) because of staff shortage, among others (Sacadura-Leite et al. 2019). However, healthcare providers working under these unfavourable conditions should still maintain work ethics and professionalism with PLHIV coinfected with TB always. It is inappropriate if nurses infringe on the rights and dignity of the patients when they seek healthcare at the PHC level. The feeling of frustration from some patients could be because of long waiting periods at the PHC or nurses embarrassing them when they default on their treatment plan. As a result, most of these patients do not come to the PHC to seek care, hence contributing to the lost-to-follow statistics. Therefore, it is imperative to establish and maintain a good nurse–patient relationship. Jarrett et al. (2019) concur that nurse–patient dynamics should instead be based on empowering these patients.

In addition, another attribute for developing a training programme identified in the review was the nurses’ confidence to effectively counsel patients considering the knowledge, counselling process and counselling content. The nurses’ knowledge about the condition under investigation, which in review is PLHIV and coinfected with TB, remains a crucial attribute to emphasise. The findings of Jarrett et al. (2019) suggest that nurses had difficulty complying with the isoniazid preventive therapy (IPT) guidelines for PLHIV in PHCs at Potchefstroom, South Africa, because of incomplete knowledge.

In this review, the importance of the nurse’s confidence to carry out effective counselling of HIV or TB people is indicated (Elvish et al. 2018; Henoch et al. 2015; Van Der Giessen et al. 2020). This review found that the nurse’s confidence during counselling goes a long way to enhancing counselling, which takes into consideration the nurse’s clinical knowledge and experience (Elvish et al. 2018; Simelane et al. 2018). Van Der Giessen et al. (2020) recommended that nurses should be confident to effectively counsel these patients by understanding the counselling process and counselling content, hence contributing to the patient’s knowledge; this is because ‘healthcare professionals lack the confidence to effectively communicate with patients with limited health literacy or migrant background’.

Additionally, beliefs about the patient’s challenging behaviour and existential and spiritual issues are some of the recommended attributes to be considered in the development of a training programme for nurses (Elvish et al. 2018; Henoch et al. 2015) to enhance adherence counselling of these patients at the PHC level. Beliefs around patients’ negative behaviour towards adherence to counselling remain a challenge; hence, the review suggested that healthcare professionals should be aware of this challenge, which would gradually improve during counselling sessions (Elvish et al. 2018). Living with a life-threatening condition like HIV and TB means living with the knowledge that the future may be limited so that existential issues might become inevitable for the ill person and their family. According to Henoch et al. (2015), spirituality is expressed through beliefs, values, traditions and practices (Puchalski et al. 2014). Therefore, this review recommended that patients’ spiritual needs should be considered when developing a training programme (Henoch et al. 2015).

Moreover, they use a conceptual model that combines the 5As: ask, alert, assess, assist and arrange. Incorporating a conceptual model of brief behaviour change counselling that combined the 5As through a guiding style derived from motivational interviews was recommended by Malan et al. (2015) and Malan et al. (2016). These counselling intervention attributes were used to guide the style into which each of the 5As would enhance patient-centredness; it served as a guiding spirit of allowing the healthcare professional to conduct effective counselling (Malan et al. 2016). The findings of a study in Hong Kong reported that using the 5As aimed to motivate community smokers to quit smoking using a brief smoking cessation; the smokers were able to quit smoking within a short period (Suen et al. 2016). Also, the finding of a study carried out in Canada on the effectiveness of tobacco dependence education versus usual or no tobacco dependence education on entry-level health professional student practice and client cessation highlighted that students’ counselling skills increased significantly following the 5As (Hyndman et al. 2019).

### Process of implementing the programme

The coordination of an interactive workshop with the nurses was one of the recommendations for developing a training programme (Hinneburg et al. 2020; Represas-Represas et al. 2013; WHO 2005). An interactive workshop significantly and positively affects members to create relationships and discussions (Hinderer & Lee 2019). This review highlighted that the interactive TB and HIV workshops should include presentations and discussions on counselling. In addition, Hinneburg et al. (2020) provided significant insight into the development of a training programme by using interactive instructional design workshops in the training programme. Including practical workshops and supervised follow-up sessions in developing a training programme, they have significantly improved the ability of primary care professionals to carry out quality patient care with the interpretation of the spirometric testing (Represas-Represas et al. 2013). According to the Southern African HIV Clinicians Society (n.d.), IPT was recommended to prevent TB in PLHIV, but the implementation of IPT in South Africa remains slow. With this challenge, the findings of Jarrett et al. (2019), which were conducted using interactive workshops with nurses and patients, suggested that the nurses believed they could not sustain their prescriptions of IPT, and though many patients intended to ask nurses about IPT, only some did. Most patients attributed their behaviour to an imbalance of patient-provider power. The findings of Jarrett et al. (2019) also reported that the interactive workshops were helpful because they addressed a gap in the knowledge while also pointing beyond to emphasising the importance of IPT for PLHIV and helping change their behaviour. Nurse–patient dynamics should be aimed at empowering PLHIV coinfected with TB; hence, interactive workshops were considered a better platform to get information from nurses and patients.

### Limitations

Incorporating the combination of the diverse nature and complexity of two research methodologies may have contributed to poor formulation of methodological rigour and inaccuracies. This review considered only articles published in English; this can be a potiential bias as there might be relevant studies published in other languages which could have enriched the outcomes of this review.

## Conclusion

Nurses’ adherence counseling skills are influenced by several factors, including their knowledge and expertise in counseling. To effectively identify patient needs, nurses must understand patients’ behavioral challenges and possess strong communication skills to build a suitable nurse-patient relationship. Nurses’ adherence counselling requires building confidence to ensure the implementation of the TB and HIV adherence counselling programme.

## References

[CIT0001] *Adherence guidelines for HIV, TB and NCDs*, viewed 04 March 2023, from https://Policy+and+service+delivery+guidelines+for+linkage+to+care%2C+adherence+to+treatment+and+retention+in+care&rlz=1C1GCEU_enZA943ZA943&sourceid=chrome&ie=UTF-8.

[CIT0002] Braun, V. & Clarke, V., 2006, ‘Using thematic analysis in psychology’, *Qualitative Research in Psychology* 3(2), 77–101. 10.1191/1478088706qp063oa

[CIT0003] Couper, I., Ray, S., Blaauw, D., Ng’Wena, G., Muchiri, L., Oyungu, E. et al., 2018, ‘Curriculum and training needs of mid-level health workers in Africa: A situational review from Kenya, Nigeria, South Africa and Uganda’, *BMC Health Services Research* 18(1), 1–12. 10.1186/S12913-018-3362-9/TABLES/5 30012128 PMC6048766

[CIT0004] Driessche, K.V., Sabue, M., Dufour, W., Behets, F. & Van Rie, A., 2009, ‘Training health care workers to promote HIV services for patients with tuberculosis in the Democratic Republic of Congo’, *Human Resources for Health* 7(1), 1–9. 10.1186/1478-4491-7-23 19291327 PMC2664786

[CIT0005] Elbireer, S., Guwatudde, D., Mudiope, P., Nabbuye-Sekandi, J. & Manabe, Y.C., 2011, ‘Tuberculosis treatment default among HIV-TB co-infected patients in urban Uganda’, *Tropical Medicine & International Health* 16(8), 981–987. 10.1111/j.1365-3156.2011.02800.x 21585625

[CIT0006] Elvish, R., Burrow, S., Cawley, R., Harney, K., Pilling, M., Gregory, J. et al., 2018, ‘“Getting to know me”: The second phase roll-out of a staff training programme for supporting people with dementia in general hospitals’, *Dementia* 17(1), 96–109. 10.1177/1471301216634926 26924840

[CIT0007] Henoch, I., Strang, S., Browall, M., Danielson, E. & Melin-Johansson, C., 2015, ‘Development of an existential support training program for healthcare professionals’, *Palliative & Supportive Care* 13(6), 1701–1709. 10.1017/S1478951515000632 26088936

[CIT0008] Higgins, S., 2016, ‘Meta-synthesis and comparative meta-analysis of education research findings: Some risks and benefits’, *Review of Education* 4(1), 31–53. 10.1002/rev3.3067

[CIT0009] Hinderer, K.A. & Lee, M.C., 2019, ‘Chinese Americans’ attitudes toward advance directives: An assessment of outcomes based on a nursing-led intervention’, *Applied Nursing Research* 49, 91–96. 10.1016/J.APNR.2019.04.003 31160144

[CIT0010] Hinneburg, J., Lühnen, J., Steckelberg, A. & Berger-Höger, B., 2020, ‘A blended learning training programme for health information providers to enhance implementation of the guideline evidence-based health information: Development and qualitative pilot study’, *BMC Medical Education* 20(1), 1–11. 10.1186/s12909-020-1966-3 PMC7079382 32183798

[CIT0011] Hyndman, K., Thomas, R.E., Schira, H.R., Bradley, J., Chachula, K., Patterson, S.K. et al., 2019, ‘The effectiveness of tobacco dependence education in health professional students’ practice: A systematic review and meta-analysis of randomized controlled trials’, *International Journal of Environmental Research and Public Health* 16(21), 4158. 10.3390/IJERPH16214158 31661922 PMC6862178

[CIT0012] Jackson, D., Hickman, L.D., Hutchinson, M., Andrew, S., Smith, J., Potgieter, I. et al., 2014, ‘Contemporary nurse whistleblowing: An integrative literature review of data-based studies involving nurses’, *Contemporary Nurse* 48(2), 240–252. 10.1080/10376178.2014.11081946 25549718

[CIT0013] Jarrett, B.A., Woznica, D.M., Tilchin, C., Mpungose, N., Motlhaoleng, K., Golub, J.E. et al., 2019, ‘Promoting tuberculosis preventive therapy for people living with HIV in South Africa: Interventions hindered by complicated clinical guidelines and imbalanced patient-provider dynamics’, *AIDS and Behavior* 24(4), 1106–1117. 10.1007/S10461-019-02675-6 PMC7085432 31549265

[CIT0014] Joint United Nations Programme on HIV/AIDS (UNAIDS), 2020, *UNAIDS data 2020*, Joint United Nations Programme on HIV/AIDS, viewed 18 April 2024, from https://www.unaids.org/en/resources/documents/2020/unaids-data. 12349391

[CIT0015] Joint United Nations Protocol on HIV/AIDS, 2017, viewed 20 June 2023, from https://www.unaids.org/sites/default/files/media_asset/20170720_Data_book_2017_en.pdf.

[CIT0016] Lei, J., Tang, B., Lu, X., Gao, K., Jiang, M. & Xu, H., 2014, ‘A comprehensive study of named entity recognition in Chinese clinical text’, *Journal of the American Medical Informatics Association* 21(5), 808–814. 10.1136/amiajnl-2013-002381 24347408 PMC4147609

[CIT0017] Long, A., Randall, T., Brettle, A.J. & Grant, M., 2022, *Evaluation tools for quantitaive research studies*, viewed 09 September 2021, from https://www.unisa.edu.au/contentassets/72bf75606a2b4abcaf7f17404af374ad/6b-evaluation_tool_for_quantitative_research_studies1.pdf.

[CIT0018] Ma, L.-L., Wang, Y.-Y., Yang, Z.-H., Huang, D., Weng, H. & Zeng, X.-T., 2020, ‘Methodological quality (risk of bias) assessment tools for primary and secondary medical studies: What are they and which is better?’, *Military Medical Research* 7, 7. 10.1186/s40779-020-00238-8 32111253 PMC7049186

[CIT0019] Madhani, F.I., Tompkins, C., Jack, S.M. & Fisher, A., 2014, ‘An integrative review of the methods used to research the prevalence of violence against women in Pakistan’, *Advances in Nursing* 2014(1), 801740. 10.1155/2014/801740

[CIT0020] Mahtab, S. & Coetzee, D., 2017, ‘Influence of HIV and other risk factors on tuberculosis’, *South African Medical Journal* 107(5), 428–434. 10.7196/SAMJ.2017.v107i5.11271 28492125

[CIT0021] Makhado, L., Davhana-Maselesele, M. & Farley, J.E., 2018, ‘Barriers to tuberculosis and human immunodeficiency virus treatment guidelines adherence among nurses initiating and managing anti-retroviral therapy in KwaZulu-Natal and North West provinces’, *Curationis* 41(1), 1–8. 10.4102/curationis.v41i1.1808 PMC6091579 29781696

[CIT0022] Malan, Z., Mash, B. & Everett-Murphy, K., 2016, ‘Evaluation of a training programme for primary care providers to offer brief behaviour change counselling on risk factors for non-communicable diseases in South Africa’, *Patient Education and Counseling* 99(1), 125–131. 10.1016/j.pec.2015.08.008 26324109

[CIT0023] Mandimika, C. & Friedland, G., 2020, ‘Tuberculosis and human immunodeficiency virus coinfection’, in P.D.O. Davies, S.B. Gordon & G.A. Rose (eds.), *Clinical tuberculosis*, pp. 267–300, CRC Press, Boca Raton, FL.

[CIT0024] Massyn, N., Barron, P., Day, C., Ndlovu, N. & Padarath, A., 2018, *District health barometer*, Health Systems Trust, viewed 18 April 2024, from https://www.hst.org.za/publications/Pages/DISTRICT-HEALTH-BAROMETER-201819.aspx.

[CIT0025] Mbunyuza, L., 2020, *Treatment adherence in TB/HIV co-infected patients in Mount Frere, Eastern Cape*, Cape Town, viewed 12 May 2021, from http://etd.uwc.ac.za/xmlui/bitstream/handle/11394/8051/mbunyuza.

[CIT0026] Moher, D., Liberati, A., Tetzlaff, J., Altman, D.G. & PRISMA Group*, T., 2009, ‘Preferred reporting items for systematic reviews and meta-analyses: The PRISMA statement’, *Annals of internal medicine* 151(4), 264–269. 19622511 10.7326/0003-4819-151-4-200908180-00135

[CIT0027] Nezenega, Z.S., Perimal-lewis, L. & Maeder, A.J., 2020, ‘Factors influencing patient adherence to tuberculosis treatment in Ethiopia: A literature review’, *International Journal of Environmental Research and Public Health* 17(15), 5626. 10.3390/IJERPH17155626 32759876 PMC7432798

[CIT0028] Ngcobo, S., Scheepers, S., Mbatha, N., Grobler, E. & Rossouw, T., 2022, ‘Roles, barriers, and recommendations for community health workers providing community-based HIV Care in Sub-Saharan Africa: A review’, *AIDS Patient Care and STDs* 36(4), 130–144. 10.1089/apc.2022.0020 35438523 PMC9057893

[CIT0029] Obeagu, E.I. & Onuoha, E.C., 2023, ‘Tuberculosis among HIV Patients: A review of prevalence and associated factors’, *International Journal of Advanced Research in Biological Sciences* 10(9), 128–134.

[CIT0030] Pascoe, M., Mahura, O. & Dean, J., 2020, ‘Health resources for South Africa: A scoping review’, *Health SA Gesondheid* 25, 1–7. 10.4102/HSAG.V25I0.1378 PMC7433232 32832107

[CIT0031] Path, T., 2009, ‘Teachers’ and learners’ experiences of learners’ writing in English first additional language: A case study of isiXhosa and Afrikaans learners’, Doctoral dissertation, University of Fort Hare.

[CIT0032] Peters, D.H., Bloom, G. & Gerdtham, U.-G., 2015, *Health systems in low- and middle-income countries: An overview of the evidence*, Cambridge University Press, Cambridge.

[CIT0033] Plowright, A., Taylor, C., Davies, D., Sartori, J., Hundt, G.L. & Lilford, R.J., 2018, ‘Formative evaluation of a training intervention for community health workers in South Africa: A before and after study’, *PLoS One* 13(9), e0202817. 10.1371/journal.pone.0202817 30248100 PMC6152868

[CIT0034] Puchalski, C.M., Blatt, B., Kogan, M. & Butler, A., 2014, ‘Spirituality and health: The development of a field’, *Academic Medicine* 89(1), 10–16. 10.1097/ACM.0000000000000083 24280839

[CIT0035] Represas-Represas, C., Botana-Rial, M., Leiro-Fernández, V., González-Silva, A.I., García-Martínez, A. & Fernández-Villar, A., 2013, ‘Short-and long-term effectiveness of a supervised training program in spirometry use for primary care professionals’, *Archivos de Bronconeumología (English Edition)* 49(9), 378–382. 10.1016/j.arbr.2013.07.005 23481409

[CIT0036] Sacadura-Leite, E., Sousa-Uva, A., Ferreira, S., Costa, P.L. & Passos, A.M., 2019, ‘Working conditions and high emotional exhaustion among hospital nurses’, *Revista Brasileira de Medicina do Trabalho* 17(1), 69. 10.5327/Z1679443520190339 32270106 PMC7138497

[CIT0037] SANAC, 2018, ‘The National Strategic Plan’, *SANAC*, viewed 15 April 2024, from https://sanac.org.za/about-sanac/the-national-strategic-plan/.

[CIT0038] Semegni, C.K., Phetlhu, D.R. & Modeste, R.R.M., 2021, ‘An integrative review of measurement instruments used to assess the stigma that affects people who use drugs’, *Sage Open* 11(1), 215824402096306. 10.1177/2158244020963067

[CIT0039] Simelane, M.L., Georgeu-Pepper, D., Ras, C.J., Anderson, L., Pascoe, M., Faris, G. et al., 2018, ‘The practical approach to care kit (PACK) training programme: Scaling up and sustaining support for health workers to improve primary care’, *BMJ Global Health* 3(Suppl 5), e001124. 10.1136/bmjgh-2018-001124 PMC6242020 30498597

[CIT0040] South African National Department of Health, 2015, *Annual report 2014/2015*, Pretoria, viewed 18 April 2024, from https://www.gov.za/sites/default/files/gcis_document/201510/health-annualreport2015.pdf.

[CIT0041] South African National Department of Health, 2016, *Human resources for health South Africa: HRH strategy for the health sector: 2016/17*, National Department of Health, Pretoria.

[CIT0042] Southern African HIV Clinicians Society, 2013, ‘Guideline for the prevention, diagnosis and management of cryptococcal meningitis among HIV-infected persons: 2013 Update’, *Southern African Journal of HIV Medicine* 14(2), a82. 10.7196/sajhivmed.930 PMC7081625 32201629

[CIT0043] Suen, Y.N., Wang, M.P., Li, W.H.C., Kwong, A.C.S., Lai, V.W.Y., Chan, S.S.C. et al., 2016, ‘Brief advice and active referral for smoking cessation services among community smokers: A study protocol for randomized controlled trial’, *BMC Public Health* 16(1), 1–7. 10.1186/S12889-016-3084-Z 27169630 PMC4866301

[CIT0044] Toronto, S.C. & Remington, R., 2020, *Nursing Informatics and the Foundation of Knowledge*, Jones & Bartlett Learning, Burlington, MA.

[CIT0045] United Nations AIDS (UNAIDS), 2016, *UNAIDS terminology guidelines – 2016*, UNAIDS, viewed 20 June 2023, from https://www.unaids.org/en/resources/documents/2016/2016_terminology_guidelines.

[CIT0046] Uwimana, J., Zarowsky, C., Hausler, H. & Jackson, D., 2012, ‘Training community care workers to provide comprehensive TB/HIV/PMTCT integrated care in KwaZulu-Natal: Lessons learnt’, *Tropical Medicine & International Health* 17(4), 488–496. 10.1111/J.1365-3156.2011.02951.X 22296235

[CIT0047] Van Der Giessen, J.A.M., Ausems, M.G., Van Den Muijsenbergh, M.E.T.C., Van Dulmen, S. & Fransen, M.P., 2020, ‘Systematic development of a training program for healthcare professionals to improve communication about breast cancer genetic counseling with low health literate patients’, *Familial Cancer* 19(4), 281–290. 10.1007/S10689-020-00176-3/FIGURES/1 32323055 PMC7497313

[CIT0048] Vera, M.G., Merighi, M.A.B., Conz, C.A., Da Silva, M.H., De Jesus, M.C.P. & González, L.A.M., 2018, ‘Primary health care: The experience of nurses’, *Revista Brasileira de Enfermagem* 71, 531–537. 10.1590/0034-7167-2016-0244 29562008

[CIT0049] viewed 22 April 2024, from https://www.hst.org.za/publications/Pages/DISTRICT-HEALTH-BAROMETER-201819.aspx.

[CIT0050] viewed 22 April 2024, from https://www.unaids.org/sites/default/files/media_asset/20170720_Data_book_2017_en.pdf.

[CIT0051] Webb Mazinyo, E., Kim, L., Masuku, S., Lancaster, J.L., Odendaal, R., Uys, M. et al., 2016, ‘Adherence to concurrent tuberculosis treatment and antiretroviral treatment among co-infected persons in South Africa’, *PLoS One* 11(7), e0159317. 10.1371/journal.pone.0159317 27442440 PMC4956242

[CIT0052] Webb Mazinyo, E., Kim, L., Masuku, S., Lancaster, J.L., Odendaal, R., Uys, M., et al., 2016, ‘Adherence to concurrent tuberculosis treatment and antiretroviral treatment among co-infected persons in South Africa, 2008–2010’, *PLoS One* 11(7), e0159317. 27442440 10.1371/journal.pone.0159317 PMC4956242

[CIT0053] Whittemore, R. & Knafl, K., 2005, ‘The integrative review: Updated methodology’, *Journal of Advanced Nursing* 52(5), 546–553. 10.1111/J.1365-2648.2005.03621.X 16268861

[CIT0054] Whittemore, R., 2005, ‘Combining evidence in nursing research: Methods and implications’, *Nursing Research* 54(1), 56–62. 10.1097/00006199-200501000-00008 15695940

[CIT0055] WHO, 2018, *Global tuberculosis report 2018 – Geneva*, WHO report, viewed 20 June 2023, from http://apps.who.int/bookorders.

[CIT0056] WHO, TB, HIV & other co-morbidities, 2020, *WHO*, viewed 05 May 2023, from http://www.who.int/tb/areas-of-work/tb-hiv/en/.

[CIT0057] World Health Organization, 2005, *World health report 2005: Working together for health*, World Health Organization, Geneva.

[CIT0058] World Health Organization, 2015, *World health report 2015: Working together for health*, World Health Organization, Geneva.

[CIT0059] World Health Organization, 2019, *World health report 2019: Working together for health*, World Health Organization, Geneva.

